# A Case of Extensive Debilitating Generalized Morphea

**DOI:** 10.7759/cureus.8117

**Published:** 2020-05-14

**Authors:** Amit Sapra, Rebecca Dix, Priyanka Bhandari, Asiya Mohammed, Eukesh Ranjit

**Affiliations:** 1 Family Medicine, Southern Illinois University School of Medicine, Springfield, USA

**Keywords:** morphea, restriction of motion, chronic pain, rare skin disease, skin biopsy, immunosuppressants, joint pain, scleroderma, joint restriction, methotrexate

## Abstract

Morphea, also known as localized scleroderma, is an uncommon idiopathic inflammatory disorder leading to the development of sclerotic plaques in the skin. The disorder preferentially affects females. The pathogenesis of morphea is not well-understood. The disorder is likely to have an autoimmune basis; environmental and genetic factors may also play a role in its etiology. Morphea has a variety of clinical presentations. Lesions of morphea typically begin as inflammatory plaques or patches that evolve into firm sclerotic lesions. Involvement may be limited to the dermis or may extend to underlying subcutaneous fat, muscle, or bone. The identification of characteristic clinical findings is often sufficient for the diagnosis of morphea. A biopsy can be a useful tool when the diagnosis is in question or to obtain information on the depth and intensity of the disease, and it should always extend at least into the subcutaneous fat. Morphea may cause joint contractures and other impairments secondary to tissue sclerosis and can be very debilitating cosmetically and functionally.

## Introduction

Morphea is a rare inflammatory disorder characterized by sclerotic manifestations of plaques of skin and is, therefore, also known as localized scleroderma. Most commonly, it affects adults but can develop at any age, and females display the disorder more than males [1].

This disorder can be distinguished from systemic scleroderma, as morphea lacks a systemic component and spares internal organs. However, morphea may be extremely debilitating. As the skin is adherent to musculoskeletal structures, the thickening of the skin can lead to severe contractures of the extremities, leading to significant loss of function, atrophy, disfigurement, and impairment [2].

We present a case of a 91-year-old Caucasian female whose symptoms started insidiously seven years ago and progressed gradually to the point that her functional impairments have her wheelchair-bound due to extensive generalized morphea. She has had treatment with a variety of pharmacological and non-pharmacological modalities, with limited success over time, and has suffered falls because of joint contractures secondary to her underlying problem. This case highlights how morphea can adversely impact a patient's activities of daily living and quality of life.

## Case presentation

Our patient is a 91-year-old Caucasian female whose symptoms started seven years ago as induration of the skin of the dorsum of feet, with "pockets" of fluid, and progressed to painful, tightened, hard lesions. The lesions then gradually spread to involve her right inner leg, left leg, and breasts. Eventually, she developed severe tightening of the skin of the lower extremities, back, and breasts, which resulted in severe pain and restriction of motion.

**Figure 1:**
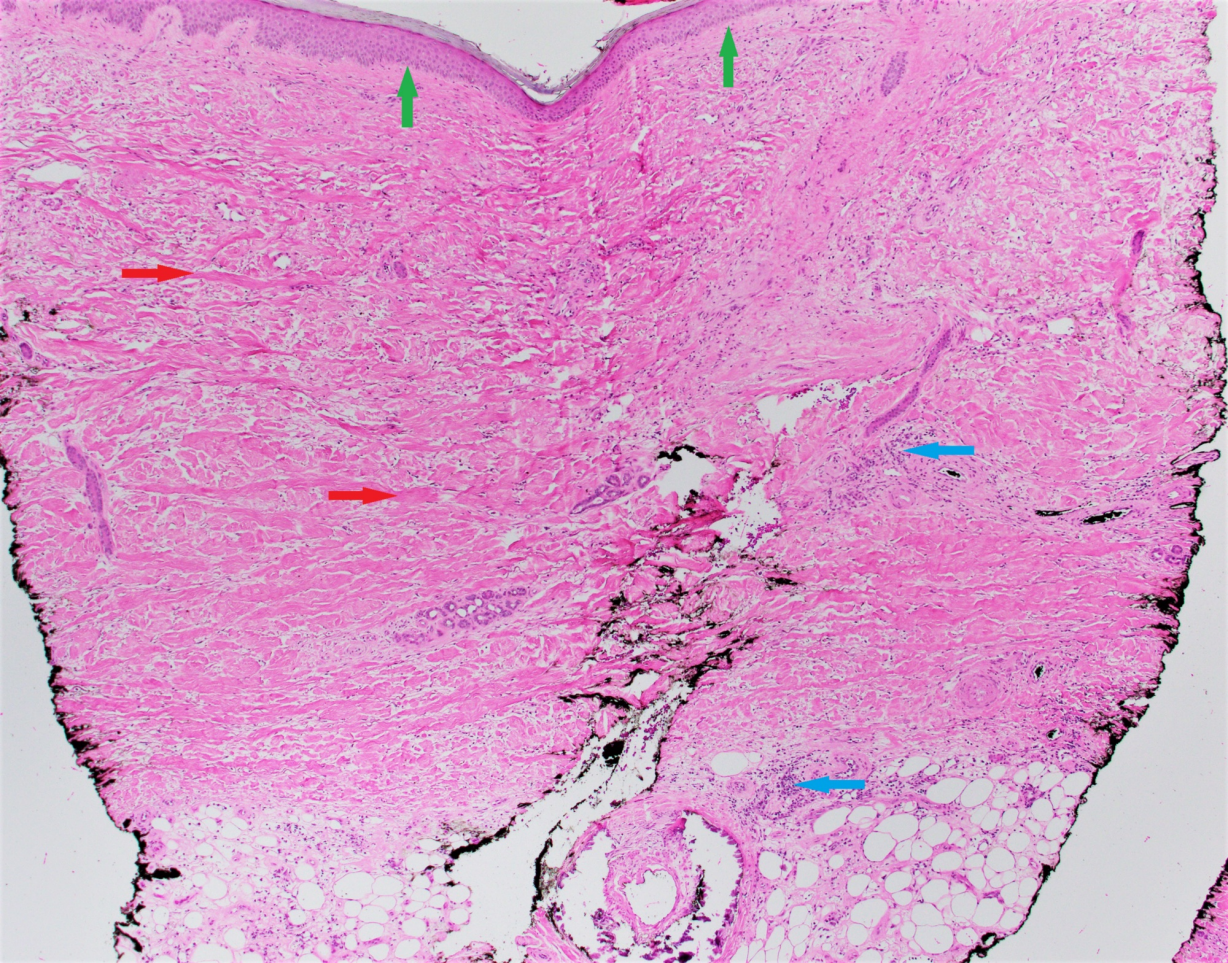
Skin biopsy showing moderate sclerosis of the dermis and subcutis (red arrows) with loss of rete ridges (green arrows) and focal chronic inflammation (blue arrows) consistent with early morphea

She was seen at the dermatology clinic at the time of onset of symptoms, and a biopsy of the skin of her right lower leg was done in late December 2014, which showed moderate sclerosis of the dermis and subcutis with loss of rete ridges and focal chronic inflammation consistent with early morphea (Figure 1).

She was started on calcipotriene cream 0.005% local application daily and tacrolimus 0.1% local application twice a day, which helped her moderately. She also underwent a trial of penicillin V for a few months as her symptoms progressed. However, she started to develop more lesions on her left lower leg, and the trial was discontinued, as it failed to help the patient.

The patient denied any mouth tightness, dysphagia, difficulty breathing, or tightness of fingers. Her hands were affected by osteoarthritis, with the involvement of the distal interphalangeal joints predominantly.

She was then referred to a rheumatology clinic, where an extensive serological workup was done. Anti-topoisomerase I antibody (Anti-Scl-70), to rule out diffuse systemic scleroderma, was found to be negative. Antinuclear antibodies (ANA) and anti-double-stranded deoxyribonucleic acid (DNA) antibodies were found to be negative and so were anti-centromere antibodies. Pulmonary function tests and renal function tests were found to be within normal limits, thus lowering our suspicion for internal disease.

The patient was started on narrow-band ultraviolet B phototherapy in early 2016 in combination with triamcinolone local ointment 0.1% to be applied to the affected areas three times a day along with calcipotriene cream 0.005% local application daily, which was continued until mid-2016 without any improvement.

Subsequently, she was started on cap mycophenolate 500 mg daily, which was later increased to twice a day dosing and noticed that her legs had "softened." This was used in addition to calcipotriene. She had noticed an increase in her bowel movements since starting mycophenolate and had episodes of stool incontinence.

In the later part of 2016, she stopped mycophenolate due to gastrointestinal side effects and hypertensive emergency (an episode of blood pressure in 200s systolic with headache, nausea, and vomiting that resolved with clonidine).

She started hydroxychloroquine 400 mg/day in late 2016 but discontinued, as the ophthalmologist noted that she already had central vision loss due to Best's disease, and it would be challenging to assess for hydroxychloroquine-induced changes in vision.

She started ultraviolet radiation treatment three times a week in 2017, which was discontinued eventually due to time constraints.

Methotrexate was started in 2018 at a 10 mg weekly dose, and the dosages were adjusted from there. She does get lab work (complete blood count and comprehensive metabolic panel) regularly while being on methotrexate. The patient reports that the tightening of the skin and discomfort has been moderately relived ever since she has started methotrexate, and it seems to have halted the progression of the disease.

She has recently been having problems with her balance, has frequently been falling because of joint contractures and other functional impairments secondary to tissue sclerosis, and is currently wheelchair-bound. She has been getting physical therapy weekly, which has helped with her symptoms.

**Figure 2:**
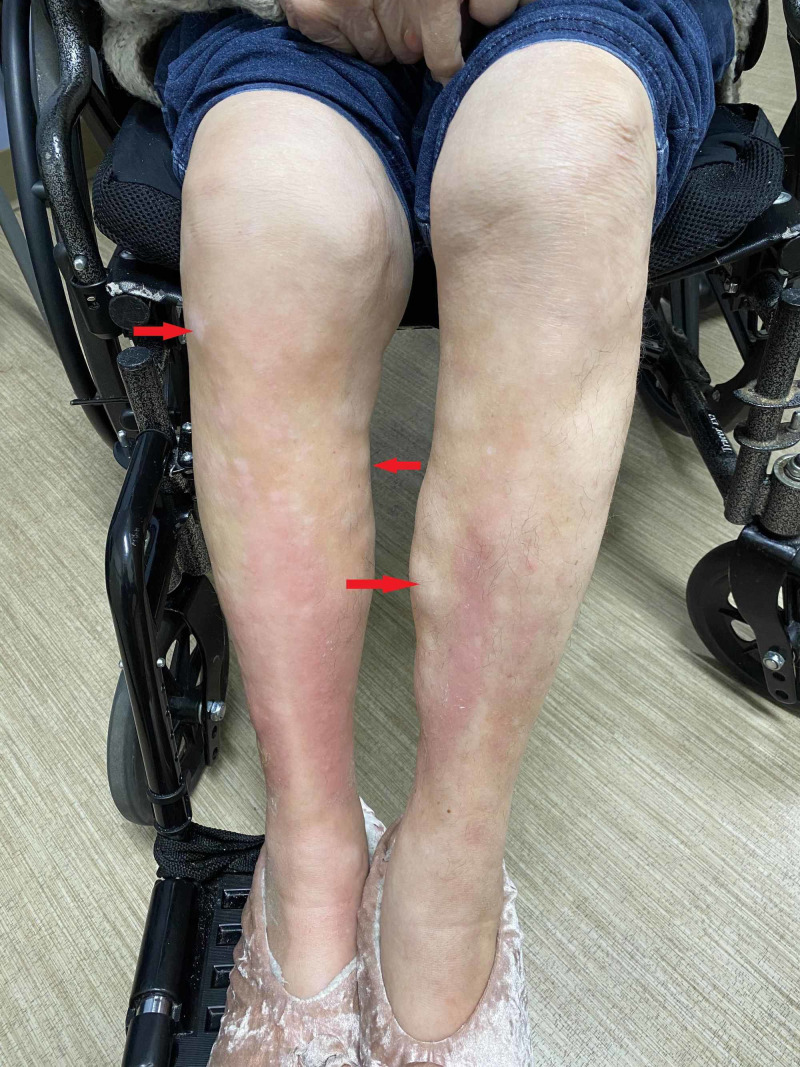
Extensive involvement of the skin of the lower extremities showing nodular areas of hypopigmentation, alopecia, and sclerosis (red arrows)

**Figure 3:**
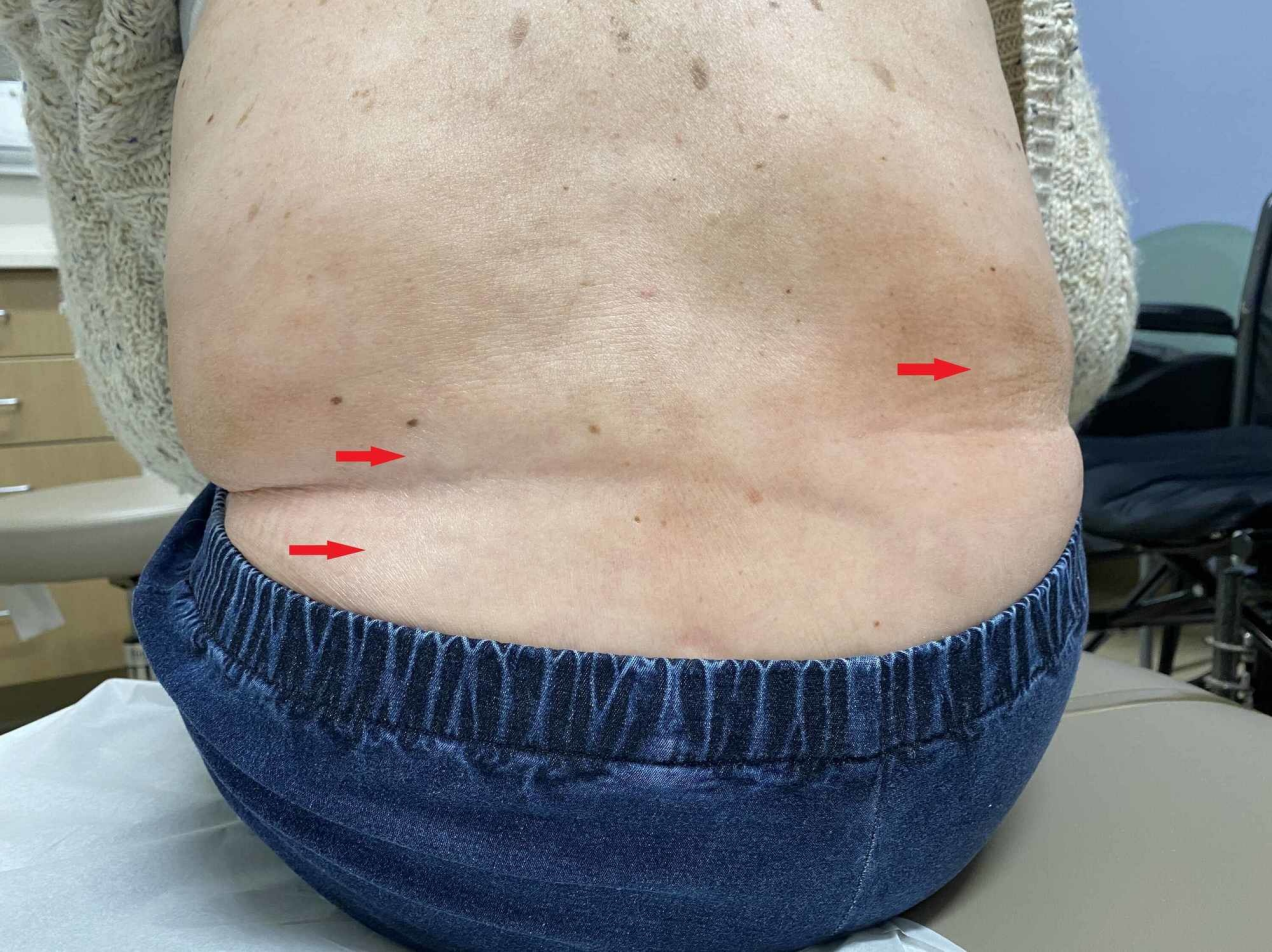
Extensive involvement of the lower back with generalized morphea (red arrows) associated with a limited range of motion in the lumbar spine and chronic pain.

**Figure 4:**
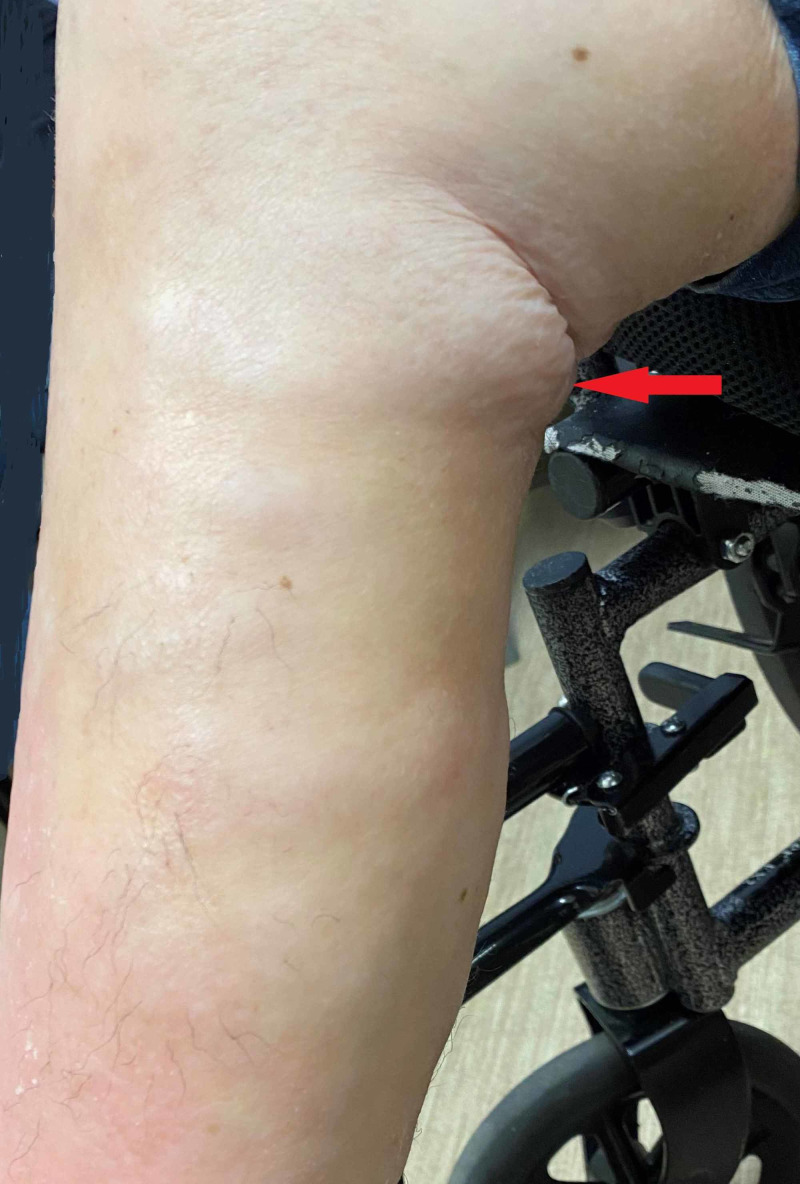
Right lower extremity showing extensive areas of thickening and hypopigmentation (red arrow), leading to the formation of contracture and restriction of knee joint motion

Figures 2-4 show the exam findings of the patient.

## Discussion

Incidence/prevalence

Morphea is a rare autoimmune disease characterized by a group of idiopathic sclerotic skin diseases and consists of a spectrum of heterogeneous disease subtypes. It ranges from solitary sclerotic lesions, which usually cause a few problems to severe complications caused by the linear subtype that may result in limb length discrepancies and joint contractures [1]. Morphea has an incidence rate between 3.4 and 27 cases per 1,00,000, and it is more common with a ratio of 2-4:1 in females to males [1,3].

Signs/symptoms

The signs and symptoms depend on the type of morphea. It is broadly classified into plaque morphea, linear morphea, generalized morphea, and mixed morphea [2]. Our patient had lesions that started as ‘red pockets’ of fluid on her right outer leg. It evolved into tight and hard skin lesions that spread to involve her right inner leg, left leg, breasts, and back. Her lower legs seemed to be experiencing worse symptoms of tightness and pain. This is in congruence with generalized morphea, defined as morphea plaques involving more than two body sites, which are often triggered by physical exercise. The onset of morphea is gradually occurring and progresses relatively fast over months. The characteristic plaques of sclerosis are slightly inflamed, pigmented, and ill-defined and occur most commonly on the trunk and extremities [2]. Joint contractures and other functional impairments secondary to tissue sclerosis can occur as the disease progresses.

Diagnosis

Scleroderma can result in the elevation of autoantibodies, with antinuclear antibodies found in 73% of patients. Of these antinuclear antibodies, anti-double-stranded make up 50%, anti-histone 47%, anti-topoisomerase 76%, and anti-centromere 12%. In generalized morphea, eosinophilia has been reported and may correlate with the severity of the disease [4]. Rheumatoid factor can also be elevated in 60% of those with scleroderma. Biopsy of the area and the histopathological features seen aid in evaluating and establishing a diagnosis of morphea and may provide information regarding the depth of involvement and degree of inflammation [5].

Pathology

Morphea and sclerosing skin diseases are characterized by excessive accumulation of the extracellular matrix in the dermis and subcutaneous tissues [6]. The histology of skin lesions in patients with scleroderma and morphea reveals the presence of early perivascular lymphohistiocytic infiltrate, which indicates early endothelial damage [6]. Due to the slow onset of the disease, the initiating events and early changes of the disease remain unknown. It is postulated that the early stages of the disease consist of vascular injury and immune dysfunction, and fibrotic changes are seen at later stages [7].

Management

Morphea is a rare disease, and because of this, there are few randomized control trials of therapeutic management. The literature to date describes the following main treatments for the disease: 1. Phototherapy with ultraviolet (UV) A-1, broad-band UVA, narrowband UVB, psoralen with UVA bath; 2. Vitamin D derivatives, which include oral calcitriol, topical calcipotriene, and calcipotriol ointments; 3. Immunosuppressors such as methotrexate, tacrolimus, chloroquine and hydroxychloroquine, colchicine, cyclophosphamide; 4. Topical steroids; and 5. Physical therapy [8].

Prognosis

The prognosis for localized scleroderma and morphea is generally good, although certain forms can have a long-term esthetic impact, especially if it affects the face and limbs [9]. Although multiple treatment modalities exist, the timing of these correctional procedures is crucial, as disease flare-up has been reported many years after disease quiescence [10]. Although the specific clinical prognosis depends on the extent of the plaques, linear deposition, and depth of the lesions, the superficial forms usually resolved within three years [11].

Morphea can potentially affect the instrumental activities of daily living (IADLs), which comprise activities like cleaning, laundry, driving, and managing finances [12].

## Conclusions

Morphea is an uncommon idiopathic inflammatory disorder leading to the development of sclerotic plaques in the skin. When this disease is diagnosed, special attention should be given to the involvement of internal organs and the association with other connective tissue diseases. As morphea may cause joint contractures and other impairments secondary to tissue sclerosis and can be very debilitating cosmetically and functionally, its early detection and the timely management of its complications are imperative. In general, the quality of life in morphea patients is significantly correlated with disease activity. Although various pharmacological and non-pharmacological methods of treatment are available, morphea is challenging and difficult to treat, and the spontaneous regression of sclerotic lesions must always be considered.
